# Screening for Novel Beneficial Environmental Bacteria for an Antagonism-Based *Erwinia amylovora* Biological Control

**DOI:** 10.3390/microorganisms11071795

**Published:** 2023-07-13

**Authors:** Guillermo Esteban-Herrero, Belén Álvarez, Ricardo D. Santander, Elena G. Biosca

**Affiliations:** 1 Departamento de Microbiología y Ecología, Universitat de València (UV), 46100 Valencia, Spain; 2 Departamento de Investigación Aplicada y Extensión Agraria, Instituto Madrileño de Investigación y Desarrollo Rural, Agrario y Alimentario (IMIDRA), 28805 Madrid, Spain; 3 Irrigated Agriculture Research and Extension Center, Washington State University, Prosser, WA 99350, USA

**Keywords:** fire blight, phytopathogenic bacteria, antagonistic activity, characterization, biocontrol

## Abstract

*Erwinia amylovora*, the bacterial species responsible for fire blight, causes major economic losses in pome fruit crops worldwide. Chemical control is not always effective and poses a serious threat to the environment and human health. Social demands for eco-sustainable and safe control methods make it necessary to search for new biocontrol strategies such as those based on antagonists. A bacterial collection from different fire blight-free Mediterranean environments was tested for antagonistic activity against Spanish strains of *E. amylovora*. Antagonistic assays were carried out *in vitro* in culture medium and *ex vivo* in immature loquat and pear fruits. Results revealed that 12% of the 82 bacterial isolates tested were able to inhibit the growth of several strains of the pathogen. Some of the isolates also maintained their antagonistic activity even after chloroform inactivation. Selected isolates were further tested *ex vivo*, with several of them being able to delay and/or reduce fire blight symptom severity in both loquats and pears and having activity against some *E. amylovora* strains. The isolates showing the best antagonism also produced different hydrolases linked to biocontrol (protease, lipase, amylase, and/or DNAse) and were able to fix molecular nitrogen. Based on this additional characterization, four biocontrol strain candidates were further selected and identified using MALDI-TOF MS. Three of them were Gram-positive bacteria belonging to *Bacillus* and *Paenarthrobacter* genera, and the fourth was a *Pseudomonas* strain. Results provide promising prospects for an improvement in the biological control strategies against fire blight disease.

## 1. Introduction

The phytopathogenic bacterial species *Erwinia amylovora* is the causative agent of the fire blight of rosaceous plants. The disease affects ornamental species of this family and fruit trees of great economic importance, such as apple, pear, or loquat, as well as some ornamental and wild species such as *Cotoneaster*, *Crataegus*, *Cydonia*, *Pyracantha*, and *Sorbus*, among others [1,2], and the disease can be easily spread by rain, wind, insects, birds, and contaminated pruning tools [3,4,5,6]. The first symptoms appear in spring when the bacterium reaches flowers and grows epiphytically on the flower stigmas. Rain and heavy dew wash the pathogen cells to the hypanthium where they enter the plant through the nectarthodes [7,8]. Additional infection routes include other natural openings in young shoots and leaves, such as stomata, hydathodes, trichomes, and/or wounds caused by wind, hail, and insects [7,9]. Once inside the host, the pathogen keeps multiplying and moving through the parenchyma and the xylem vessels, establishing systemic infections [10,11]. Every plant organ is susceptible to infection, although young tissues are more susceptible than lignified, older tissues [4,9]. The main fire blight symptoms include necrosis and secretion of ooze droplets on the affected organ’s surface [9]. These ooze droplets contain high concentrations of the pathogen, and their dissemination to susceptible plant tissues contributes to secondary infections of flowers and green tissues [9,12,13]. Affected shoots can curve in a characteristic shepherd’s crook shape. In some cases, infected trees or organs remain asymptomatic, constituting sources of inoculum that may pass unnoticed [14,15]. When the pathogen reaches perennial tissues of branches, the trunk and/or the rootstock induce the formation of cankers [9]. Different environmental factors and host resistance to the infecting strain determine the survival of *E. amylovora* in cankers [16] during winter. In spring, with the renewal of the host growth, *E. amylovora* cells multiply in active cankers, and ooze droplets are released to the canker surface serving as the primary inoculum source for a new infection cycle [4,13].

Due to the devastating nature of *E. amylovora*, the pathogen is considered a quarantine pest of protected areas in the European Union (EU), Russia, and different Mediterranean countries belonging to the European and Mediterranean Plant Protection Organization [17,18]. As fire blight produces significant economic losses worldwide [19,20], the protection of susceptible crops is a key factor for agricultural sustainability and food security [21]. Among the most common preventive and cultural control methods for the disease are pruning infected branches, avoiding sprinkler irrigation, and reducing the amount of nitrogen fertilizers, as well as preventing the introduction of sensitive plant material with latent infections. However, when these measures are not enough, physical, chemical, and/or biological control strategies are needed. In general, the most effective control methods against *E. amylovora* are based on the use of antibiotics such as streptomycin or copper compounds that are applied in the field during the flowering season. Although these are effective, continued use can lead to the development of resistance by the pathogen [22]. This, together with growing concern about public health problems derived from their presence in food, does not make them viable in the long term [23]. Regarding cupric compounds such as copper hydroxide or copper sulfate, their application can have phytotoxic effects, in addition to accumulating in the environment, so their use is being reduced thanks to the social demand for more sustainable, safe, and ecological methods [24]. In the EU, the use of antibiotics is prohibited in agriculture, and the use of copper-based compounds is highly restricted [14].

An alternative to traditional agrochemical compounds is the development of effective and ecological biological control methods such as those based on the use of microorganisms [25]. These biocontrol agents (BCAs) can be competing microorganisms, such as bacteria, yeasts and filamentous fungi [24], or predators, such as natural bacteriophage viruses of the pathogen [26]. With respect to *E. amylovora* biological control with antagonistic microorganisms, the suppression of the pathogen cells during the flowering period is an important factor in preventing their systemic spread [27]. In this sense, both bacteria and yeasts have been marketed as BCAs against this pathogen. These compete for nutrients and space [28], but they can also produce metabolites, bioactive compounds, and lytic enzymes that inhibit the growth of the pathogen [29,30,31] or induce localized resistance against the pathogen [32]. The most widely used commercialized bacteria as BCAs against *E. amylovora* are *Pantoea agglomerans* (strains C9-1 and P10C) [33], *Pseudomonas fluorescens* (strain A506) [34], and *Bacillus subtilis* (strain QST713) [35]. However, the efficacy of these antagonistic bacteria is closely linked to environmental conditions, so it is still necessary to search for new bacteria with biocontrol potential against *E. amylovora* adapted to specific climate conditions where susceptible *E. amylovora* hosts are present.

In this work, the potential *in vitro* and *ex vivo* antagonistic activity against *E. amylovora* of a collection of environmental bacterial isolates from fire blight-free Mediterranean environments in Spain was evaluated, and those with the greatest antagonistic potential were characterized. The bacterial isolates considered the best candidates as BCAs against *E. amylovora* were identified.

## 2. Materials and Methods

### 2.1. Bacterial Strains and Culture Conditions

A collection of 82 bacterial isolates from various environmental samples (rainwater, river water, or soil) from several locations of the Valencian Community (Spain), with a mild Mediterranean climate, were grown and subsequently purified from Nutrient Agar (NA) at 28 °C for 48 h prior to being initially assessed for antagonistic activity against *E. amylovora*. Bacterial antagonism was evaluated using four *E. amylovora* strains isolated from diverse host plants and geographical origins: the European reference strain CFBP 1430 and three isolates from different locations in Spain (Table 1). All *E. amylovora* strains were routinely cultured in NA at 28 °C for 48 h and were handled under Biosecurity Level 2 conditions. Re-isolation of *E. amylovora* from *ex vivo* assays was performed in the semi-selective CCT medium [36]. In all cases, the pure cultures of the strains were cryopreserved at −80 °C in 25% (*v*/*v*) glycerol.

### 2.2. In Vitro Antagonistic Activity: Cross-Streak and Double-Layer Agar Assays

The potential antagonistic activity of the 82 bacterial isolates was initially tested by a modified cross-streak method [40]. Cultured cell suspensions were adjusted to O.D._600_ 0.1 ± 0.01 and diluted one-tenth for *E. amylovora* [10^7^ CFU (colony-forming units)/mL] and O.D._600_ 0.2 ± 0.02 (10^8^ CFU/mL) for potential antagonistic candidates, respectively, in sterile 10 mM Phosphate Buffered Saline (PBS) with pH = 7.2. Briefly, an adjusted suspension of each candidate was individually inoculated, by drawing a line on NA plates and incubated at 28 °C for 48 h. Then, all four pathogenic *E. amylovora* strains were co-inoculated on the same agar plate on a straight perpendicular line. Each candidate was assessed in duplicate against the four *E. amylovora* strains. For control treatment, *E. amylovora* strains were cultured alone, and all plates were incubated at 28 °C for 48 h. Based on these assays, the 10 candidates with better results were selected for further analysis. To determine whether these candidates maintained their antagonistic activity after their biological inactivation, the same assay was repeated including a 15-min chloroform exposure step of the antagonists before streaking the *E. amylovora* strains. When the ability of the candidates to inhibit *E. amylovora* growth is due to the production of antimicrobial substances diffused into the culture medium, they maintain their activity after cell inactivation. When the ability of the candidates to inhibit *E. amylovora* growth is due to competition for space and/or nutrients in the medium, the inhibitory effect is no longer observed after cell inactivation.

The antagonistic activity was also assayed by a modified version of the double-layer agar method [41] to observe *E. amylovora* growth inhibition halos around the growth area of the tested isolate. In short, 10 μL of a suspension of each candidate adjusted to 10^8^ CFU/mL was inoculated onto the center of the plate and then incubated at 28 °C for 48 h. Afterwards, a second layer of top agar was added, which was prepared by mixing 4.5 mL of the agar, melted, and tempered, with 0.5 mL of the suspension of the *E. amylovora* strain adjusted to 10^8^ CFU/mL and incubated at 28 °C for 48 h. The appearance of a halo around the candidate was considered positive for antagonism against *E. amylovora*.

### 2.3. Ex Vivo Antagonistic Activity: Assays on Detached Fruit

*Ex vivo* assays were carried out in immature fruits of two sensitive *E. amylovora* host plant species: loquats (*Eriobotrya japonica* cv. Tanaka) of 2.3–2.8 cm diameter, and pears (*Pyrus communis* cv. Williams) of 2.1–2.7 cm diameter. Detached fruits were thoroughly surface-disinfected by immersion in 2% NaClO (*v*/*v*) for 5 min, rinsed three times with sterile distilled water, and carefully left to dry on sterile filter paper in the hood. Before inoculation, three equidistant wounds were made on each fruit with a sterile pipette tip, as described elsewhere [42].

Each wound was inoculated with 10 µL of the suspension of each candidate (ca. 10^6^ CFU/wound) for 24 h before adding another 10 µL of *E. amylovora* suspension (ca. 10^5^ CFU/wound). Fruits were incubated at 28 °C and in high relative humidity in wet chambers, as described previously [42]. Negative and positive fire blight symptom controls inoculated with PBS and *E. amylovora*, respectively, as well as controls inoculated only with the antagonists separately, were also included in the assays. Additionally, fruits inoculated with *Enterobacter cancerogenus* T2-27 were used as positive antagonistic activity control [43]. Each antagonistic strain was tested in four or three replicate fruits, in two independent experiments.

After inoculation, fruits were monitored for fire blight symptom development over time. The “Efficacy” of fire blight symptom control [E (%)] was achieved by the potential candidates, and the “Severity” of the disease [S (%)] was calculated as follows:

E (%) = ((ic − it)/T) · 100

$$S\;(\%)=\sum_{it\to 1}^{it}\left(\frac{{SI}_{it}}{T\cdot SImax}\right)\cdot 100$$

where “ic” is the number of wounds inoculated with PBS, “it” is the number of symptomatic wounds in the treatment, “T” is the total number of inoculated wounds, “SI_it_” is the symptom Severity Index of a given inoculated wound, rated according to a visual scale of the symptoms from 0 to 3 as described by [44], and “SI_max_” is the maximum SI value; in this case, the maximum value is 3. These two parameters, “E” and “S”, were evaluated at 5, 7, and 9 days post-inoculation (dpi) in Tanaka loquats and at 3, 5, and 7 dpi in Williams pears, respectively [45].

Following *ex vivo* assays, re-isolations of *E. amylovora* from the tested immature fruits were performed on a semi-selective CCT medium, which was PCR-identified by amplification of the 16S rRNA gene, using G1f/G2r primers as described [46].

### 2.4. Characterization of Candidates with Antagonistic Activity

#### 2.4.1. Exoenzymatic Activities

The ability to hydrolyze proteins was detected as transparent halos around the colonies in Casein Agar medium [NA supplemented with 10% casein (*v*/*v*)] according to [47]. Clearing halos around the growth zone are indicative of protease production. DNase activity was assayed in DNase Agar medium (DNA 2 g/L, casein peptone 1.5 g/L, soy peptone 0.5 g/L, NaCl 5 g/L, agar 15 g/L, pH 7.2–7.4) [48]. Plates incubated at 28 °C for 48 h were filled with 1 M HCl to reveal the presence of transparent halos around the growth area due to DNA degradation. Cellulase production was assayed in Cellulose Agar medium (0.5 g/L KH_2_PO_4_, 0.25 g/L MgSO_4_, 2 g/L cellulose, 2 g/L gelatin, 15 g/L agar, pH 7.0) as described by [49]. Plates incubated at 28 °C for 48 h were washed with distilled water and were flooded with 0.1% (*w*/*v*) Congo-Red solution in 20% ethanol for 15 min. The liquid was removed, and the plates were decolorized by adding 1 M NaCl solution to reveal the activity. The presence of halos on the discolored medium indicates cellulose hydrolysis. Amylase production was assayed in Starch Agar medium (beef extract 3 g/L, NaCl 2 g/L, dissolvable starch 20 g/L, agar 12 g/L) [50]. To reveal starch degradation, a Lugol solution containing 0.1 M I_2_ and 0.2 M KI was used, which only reacts with starch, giving rise to a purple coloration. The amylolytic activity was differentiated by the appearance of transparent halos. Lipase production was assessed in Tween 80 Agar medium (peptone 10 g/L, NaCl 5 g/L, CaCl_2_ 0.1 g/L, Tween 80 10 g/L, agar 15 g/L) [47]. Degradation of Tween 80 will release free fatty acids, which will react with the Ca^+2^ ions, generating opaque calcium salts, which precipitate around the lipase-producing colonies.

#### 2.4.2. Nitrogen Fixation

This activity was assayed by streaking the candidate strains into nitrogen-deficient Norris’ minimal medium (1 g/L K_2_HPO_4_, 0.2 g/L MgSO_4_·7H_2_O, 1 g/L CaCO_3_, 0.2 g/L NaCl, 0.1 g/L FeSO_4_·7H_2_O, 0.005 g/L Na_2_MoO_4_·2H_2_O, 10 g/L glucose, 15 g/L agar, pH 7.2) [51]. Bacterial growth in this medium was considered indicative of the molecular nitrogen-fixing capacity.

### 2.5. Identification of Bacterial Strains by MALDI-TOF MS

After the characterization of the bacterial isolates, a selection of the candidates with the best biocontrol potential, supported by the presence of some additional biotechnological activities, was performed for subsequent identification. This was carried out by using Matrix Assisted Laser Desorption Ionization-Time of Flight Mass Spectrometry (MALDI-TOF MS) analyses, following the protocol recommended by Bruker Daltonics [52], at the Spanish Type Culture Collection (CECT) of the Universitat de València (Spain). Protein profiles were obtained for each of the bacterial isolates from their ribosomal proteins and were subsequently compared with the protein profiles present in reference databases. Purified cultures grown in NA and incubated at 28 °C for 48 h were prepared for the analyses. The Microflex L20 Mass Spectrometer and the BioTyper 3.1 program were used for data obtention and processing.

### 2.6. Statistical Analysis

In the *ex vivo* antagonistic assays, a two-way analysis of variance (two-way ANOVA) was used to compare results obtained with the bacterial candidates and the positive controls (*E. amylovora* CFBP 1430, IVIA 1554, IVIA 1614.2 and IVIA 1892.1) in two independent experiments carried out under the same conditions. The statistical analysis of the data was performed with the GraphPad Prism 9 software. In all analyses, a *p* value < 0.05 was considered statistically significant. All results were represented as the mean ± the standard deviation (SD).

## 3. Results and Discussion

In many parts of the world, the control methods in crop protection against *E. amylovora* are frequently based on the preventive but not curative use of antibiotics such as streptomycin and/or copper-based compounds. However, the associated emerging antibiotic resistance has led the EU to ban their use in plant protection products [30,53], and the phytotoxicity issues related to the application of copper have resulted in major restrictions on its use [24,54]. The importance of finding an environmentally friendly and effective control mechanism against fire blight due to the lack of efficient phytosanitary control measures to stop its dissemination makes *E. amylovora* a pathogen of great concern worldwide [55]. In recent years, biological control with microbial antagonists has been considered a powerful and eco-friendly alternative [56]. In this panorama, the aim of this work was to evaluate the potential antagonistic activity of a collection of environmental bacterial isolates against *E. amylovora*.

### 3.1. E. amylovora-Free Environments Can Be a Source of E. amylovora Antagonists

Based on the *in vitro* cross-streak assay, 10 of the 82 bacterial isolates tested were selected as good candidates for further characterization. These were candidates UV-17, UV-18, UV-19, UV-20, and UV-41, which inhibited the growth of all the four tested *E. amylovora* control strains, and candidates UV-9, UV-12, UV-30, UV-59, and UV-60, which inhibited the growth of between one and three of the tested *E. amylovora* strains (Table 2).

Candidates UV-12 and UV-30 were not able to maintain their antagonistic activity against the pathogen after their inactivation with chloroform. This indicates either potential contact-dependent antagonistic activity, quick inactivation of released antimicrobial compounds under the assayed conditions, and/or active modification of the medium pH while feeding on the media, although more tests are required to confirm these possibilities [57]. Most of the antagonist candidates listed in Table 2 showed *E. amylovora* inhibitory effects with and without inactivation, suggesting the production of stable antimicrobial substances that diffused through the medium and remained active during the assayed period.

**Figure 1:**
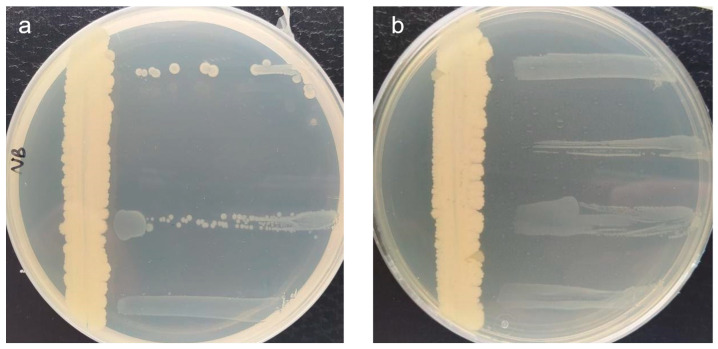
Representative images of *in vitro* antagonism assays performed by the cross-streak method with an active (**a**) and a chloroform-inactivated (**b**) biocontrol candidate against *Erwinia amylovora*. The candidate was inoculated vertically. The *E. amylovora* strains were perpendicularly inoculated 48 h after the candidate (from top to bottom: CFBP 1430, IVIA 1554, IVIA 1614.2, and IVIA 1892.1). In this case, the candidate lost its potential biocontrol activity against some *E. amylovora* strains after chloroform inactivation, suggesting that the growth inhibitory activity may be due more to competition for space and/or nutrients in the medium than to the production of antimicrobial substances.

Based on the results, candidates UV-12, UV-17, UV-18, UV-19, UV-20, UV-30, and UV-41 were selected due to their stronger antagonistic activity [higher activity values (++/+++)] against three or more *E. amylovora* control strains, despite some of them (UV-12 and UV-30) completely lost their function after chloroform inactivation [24]. A strong antagonistic activity against this pathogen has already been described by the use of plant-associated antagonists that compete for the scarce nutrients in the medium [43]. Representative pictures of the cross-streaking method results are shown in Figure 1.

*In vitro* antagonistic activity of the bacterial isolates was also tested by the double-layer agar method as described by [41]. However, inhibition halos of *E. amylovora* could not be quantified, then no comparative readings could be made using this method.

### 3.2. Ex Vivo Antagonistic Activity on Detached Fruit

The biocontrol potential of the ten candidates selected *in vitro* was also assessed *ex vivo* on immature fruits of two host species susceptible to *E. amylovora*: Tanaka loquats and Williams pears.

#### 3.2.1. Antagonistic Activity of the Candidate Strains against Fire Blight in Loquats

**Figure 2:**
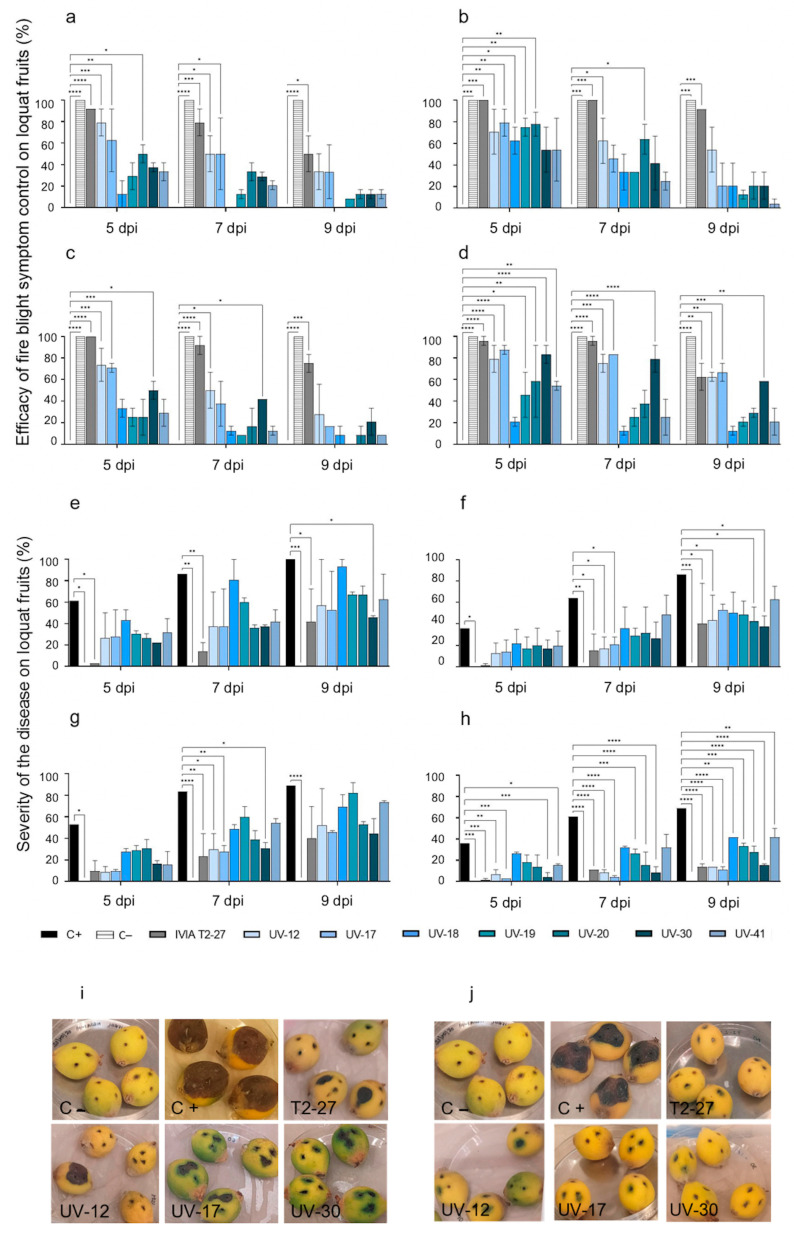
Antagonistic activity on Tanaka loquats of the selected candidates against *Erwinia amylovora* strains at 5, 7, and 9 dpi. From left to right and top to bottom: efficacy of fire blight symptom control against CFBP 1430 (**a**), IVIA 1554 (**b**), IVIA 1614.2 (**c**), and IVIA 1892.1 (**d**); disease severity against CFBP 1430 (**e**), IVIA 1554 (**f**), IVIA 1614.2 (**g**), and IVIA 1892.1 (**h**). Mean values of two independent experiments ± standard deviation are shown. The asterisks point out significant differences compared to the positive control (* *p* value < 0.05, ** *p* value < 0.01, *** *p* value < 0.001, **** *p* value < 0.0001). Representative pictures of symptomatology on loquats at 9 dpi in assays with *E. amylovora* CFBP 1430 (**i**) and IVIA 1892.1 (**j**). From left to right and top to bottom: C− (PBS), C+ (*E. amylovora strain*), strain *E. cancerogenus* IVIA T2-27, and candidates UV-12, UV-17, and UV-30.

An initial test was conducted as a set-up of the experiment. Candidates UV-9, UV-59, and UV-60, which had already been discarded for their low *in vitro* activity (Table 2), were also discarded for their low *ex vivo* activity against *E. amylovora*. Two independent experiments were conducted on immature loquats with the new selection of candidates (UV-12, UV-17, UV-18, UV-19, UV-20, UV-30, UV-41, and the infection inhibition control *E. cancerogenus* IVIA T2-27) and 1/10 dilutions of the *E. amylovora* control strains. Results are summarized in Figure 2. Regarding Tanaka loquats inoculated with the positive *E. amylovora* inhibition control IVIA T2-27, they showed 76% control of symptom development, the highest in all cases. Further, in the few inoculated wounds showing symptoms, the severity was also the lowest throughout the experimental period. The results confirmed the antagonistic activity of strain IVIA T2-27 from loquat microbiota against the *E. amylovora* strains [43].

With respect to fruits inoculated with the selection of candidates, significant antagonistic activity was confirmed at 5 dpi. However, 100% efficacy was not achieved in any case, and infection control decreased along with incubation time (Figure 2a–d). Some candidates were able to notably reduce the severity of the symptoms compared to the positive infection controls. In general, fruits inoculated with candidates UV-12, UV-17, and UV-20 achieved greater efficacy against all *E. amylovora* strains, with significant differences from the positive control at 5 dpi in all cases: candidate strain UV-12 showed a control efficacy ranging from 70–80%, depending on the assayed *E. amylovora* strain. Candidates UV-17 and UV-20 showed control efficacies of 65–85% and 50–75%, respectively. These strains also showed the greatest capacity to reduce symptom development in the tested loquats throughout the experiment. The efficacy of candidate UV-30 in controlling symptom development and severity was also significantly better than untreated controls when assayed against *E. amylovora* IVIA 1554 and IVIA 1892.1. The lowest disease severity was observed in fruits inoculated with candidates UV-12, UV-17, and UV-30 regardless of the tested *E. amylovora* strain (Figure 2e–h).

In the *E. amylovora*-inoculated fruits (positive fire blight controls), the disease severity at 5 dpi varied according to the pathogenic strain tested: 61% for CFBP 1430 (Figure 2e), 53% for IVIA 1614.2 (Figure 2g) and 36% for IVIA 1554 (Figure 2f) and IVIA 1892.1 (Figure 2h). Symptom development was progressive, and at 9 dpi severity was 100% for CFBP 1430, 86% for IVIA 1554, 89% for IVIA 1614.2, and 69% for IVIA 1892.1. No fire blight symptoms were observed throughout the experiment in the fruits inoculated with PBS (Figure 2) or with the antagonists alone.

Representative pictures of infection and symptoms on the immature Tanaka loquats from these *ex vivo* antagonistic assays are also shown in Figure 2i,j.

#### 3.2.2. Antagonistic Activity of the Candidate Strains against Fire Blight in Pears

**Figure 3:**
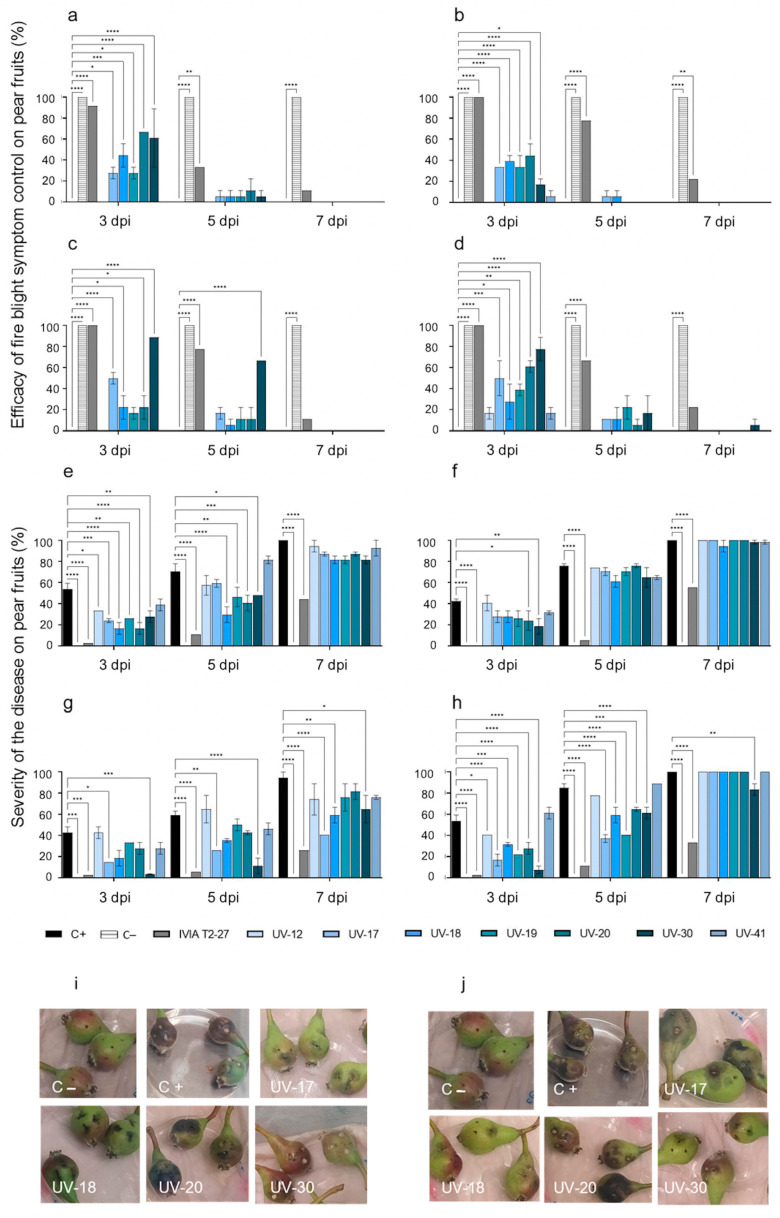
Antagonistic activity on Williams pears of the selected candidates against *Erwinia amylovora* strains at 3, 5, and 7 dpi. From left to right and top to bottom: efficacy of fire blight symptom control against CFBP 1430 (**a**), IVIA 1554 (**b**), IVIA 1614.2 (**c**), and IVIA 1892.1 (**d**); disease severity against CFBP 1430 (**e**), IVIA 1554 (**f**), IVIA 1614.2 (**g**), and IVIA 1892.1 (**h**). Mean values of two independent experiments ± standard deviation are shown. The asterisks point out significant differences compared to the positive control (* *p* value < 0.05, ** *p* value < 0.01, *** *p* value < 0.001, **** *p* value < 0.0001). Representative pictures of symptomatology on pears at 3 dpi in assays with *E. amylovora* CFBP 1430 (**i**) and IVIA 1892.1 (**j**). From left to right and top to bottom: C− (PBS), C+ (*E. amylovora* strain), and candidates UV-17, UV-18, UV-20, and UV-30.

Due to the rapid onset of fire blight symptoms observed in immature pear fruits, the incubation time had to be reduced to 7 days, taking readings at 3, 5, and 7 dpi. Results are shown in Figure 3. Fruits inoculated with *E. amylovora* 24 h after their inoculation separately with the selection of candidates showed significant control of symptom development at 3 dpi (Figure 3a–d). Fruits inoculated with candidates UV-17, UV-20, and UV-30 showed the best efficacy against *E. amylovora* strains (Figure 3a–d). With a value of up to 89% in fruits inoculated with *E. amylovora* IVIA 1614.2 at 3 dpi and 67% at 5 dpi, UV-30 showed the best antagonistic activity among the evaluated candidates (Figure 3c). To a lesser extent, some efficacy was achieved with candidate UV-19, mainly against IVIA 1614.2 and IVIA 1892.1, controlling symptom development in 50% of the inoculated fruits at 3 dpi (Figure 3c,d, respectively).

Despite the relatively good disease control efficacy provided by some of the candidate strains at 3 dpi, a drastic drop in the efficacy took place at 5 dpi (Figure 3a–d). By 7 dpi, fruits inoculated with candidate UV-30 against *E. amylovora* IVIA 1892.1 were the only ones with some efficacy (6%) (Figure 3d).

The disease severity (%) at 3 dpi in *E. amylovora*-inoculated fruits varied depending on the tested strain (Figure 3e–h): 48% for CFBP 1430 (Figure 3e), 41% for IVIA 1554 (Figure 3f), 37% for IVIA 1614.2 (Figure 3g), and 59% for IVIA 1892.1 (Figure 3h). Symptom development advanced rapidly, and at 7 dpi, the severity was 94% for IVIA 1614.2 and 100% for the other three *E. amylovora* strains. No fire blight symptoms were observed throughout the experiment in the fruits inoculated with PBS (Figure 3e–h) or with the antagonists alone. The lowest disease severity was observed in the fruits inoculated with candidate UV-30 against *E. amylovora* strains IVIA 1554, IVIA 1614.2, and IVIA 1892.1 (Figure 3f–h) and candidates UV-18 and UV-19 against *E. amylovora* strain CFBP 1430 (Figure 3e), with significant differences with the positive control in all cases at 3 dpi. The low severity observed in fruits inoculated with candidate UV-17 against IVIA 1614.2 and IVIA 1892.1 was also remarkable (Figure 3g,h, respectively).

Regarding the disease control efficacy on fruits inoculated with the positive antagonistic activity control strain *E. cancerogenus* IVIA T2-27, it was 89–100% at 3 dpi, the highest in all cases (Figure 3a–d), and the severity was also the lowest during all incubation time (Figure 3e–h). These results confirmed the antagonistic activity of strain *E. cancerogenus* IVIA T2-27 from loquat microbiota against the assayed *E. amylovora* strains, according to previous studies [40].

Representative pictures of infection and symptoms on the immature Williams pears from these *ex vivo* antagonistic assays are also shown in Figure 3i,j.

**Figure 4:**
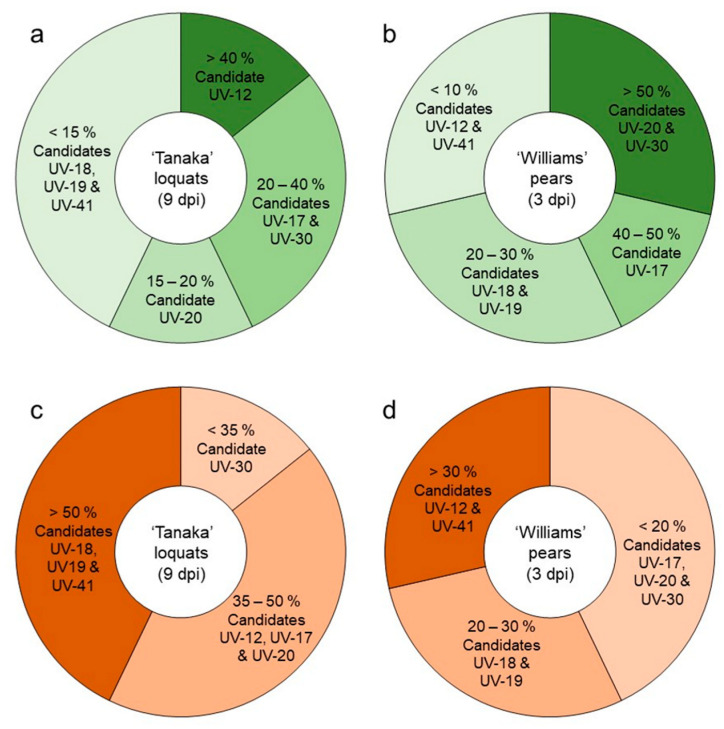
Summary of the selection of biocontrol candidates assessed *ex vivo* against all four *Erwinia amylovora* strains on immature fruits. The green scale of the upper pie charts represents the efficacy of infection inhibition (E %) on Tanaka loquats (**a**) and Williams pears (**b**). The red scale of the lower pie charts represents the severity of infection (S %) on Tanaka loquats (**c**) and Williams pears (**d**). The readings were based on the E or S values obtained by the candidates on the fruits inoculated with *E. amylovora* strains, where the differences in the intensity of the color scale were related to the obtained value (E or S %). Higher values displayed more intense color.

Altogether, results revealed candidate UV-12 as the most effective in Tanaka loquats at 9 dpi (above 40%), followed by UV-17 and UV-30 (20 to 40%) and UV-20 (15 to 20%) (Figure 4a). On the other hand, the most effective candidates in Williams pears at 3 dpi were UV-20 and UV-30 (above 50%), followed by UV-17 (40 to 50%) and candidates UV-18 and UV-19 to a lesser extent (20 to 30%) (Figure 4b). Regarding the disease severity, UV-30 was the candidate that most reduced the appearance of symptoms in Tanaka loquats at 9 dpi (below 35%), followed by candidates UV-12, UV-17, and UV-20 (35 to 50%) (Figure 4c). Candidates UV-30, UV-17, and UV-20 were those that better controlled the appearance of symptoms in Williams pears (below 20%), followed by UV-18 and UV-19 (20 to 30%) (Figure 4d).

This work highlights the influence the environment may have on antagonism. Potential biocontrol agents may not behave the same in a controlled space as shown in *in vitro* assays, as in *ex vivo* assays, where many variables (available nutrients, pH, humidity, and temperature conditions) and even the microbiota of the plant material itself can have an influence, making it difficult to standardize. In addition, the differences obtained among potential biocontrol candidates and *E. amylovora* strains on immature loquats (cv. Tanaka) and pears (cv. Williams) point out the importance of performing *ex vivo* assays on plant material from different species.

### 3.3. Characterization of Candidates with Antagonistic Activity

Results of the exoenzymatic activity and nitrogen-fixing activity tests are shown in Table 3. All candidates exhibited at least two of the activities tested, and only one (candidate UV-9) was positive for all the activities. After 96 h of growth, they showed that amylase, protease, and DNase activities were the most common, being positive in eight (candidates UV-9, UV-17, UV-18, UV-19, UV-20, UV-41, UV-59, and UV-60), seven (candidates UV-9, UV-12, UV-17, UV-18, UV-19, UV-20, and UV-41) and seven (candidates UV-9, UV-12, UV-17, UV-18, UV-19, UV-59, and UV-60) of the ten candidates, respectively. Lipase activity was less frequent, being present in only four candidates (UV-9, UV-12, UV-30, and UV-41). Candidates UV-9 and UV-41 were also the only ones positive for cellulase activity.

The nitrogen-fixing activity was the growth-promoting activity tested in this work. As shown in Table 3, candidates UV-12, UV-17, UV-20, and UV-30 were able to fix nitrogen at 48 h. Candidates UV-18 and UV-19 showed nitrogen-fixing activity at 96 h. Candidates UV-9, UV-41, UV-59, and UV-60 did not show nitrogen-fixing capacity throughout the whole assay.

A second selection of candidates was made based on the results: candidates UV-12, UV-17, UV-20, and UV-30 displayed the best efficacy against *E. amylovora* and the lowest disease severity on immature fruits in general. They also showed earlier nitrogen-fixing activity than other candidates and were negative for cellulase activity, which could interfere with planta assays due to its ability to degrade plant material, which has been described in many plant pathogens as a virulence factor [58].

### 3.4. Identification of Bacterial Strains by MALDI-TOF MS

The selected candidates with the highest biocontrol potential, based on the *in vitro* and *ex vivo* assays, also supported by the presence of some additional biotechnological activities, were identified by the MALDI-TOF MS methodology (Table 4). Candidates UV-17 and UV-30 were identified as *Paenarthrobacter aurescens* and *Pseudomonas moraviensis*, respectively, and candidates UV-12 and UV-20 as species within the genera *Bacillus* and *Paenarthrobacter*, potentially *B. simplex* and *P. nicotinovorans*, respectively. All three genera correspond to bacteria described in different environmental samples as plant growth-promoting rhizobacteria [57,59].

Results support the existing body of literature that places *Bacillus* spp. As one of the most studied species for biocontrol. Not only a multitude of direct antagonistic mechanisms against plant pathogens, including hydrolytic enzyme and antibiotic production or competition for nutrients and space but also excellent plant growth-promoting and disease-reducing properties of plants caused by both phytopathogenic fungi and bacteria have been reported [60,61]. *B. simplex* has previously been described with a wide range of activities related to biocontrol potential, supported by the exoenzymatic production and nitrogen-fixing abilities observed in candidate UV-12 [62,63].

Some species of the genus *Paenarthrobacter* have been reported to have remarkable metabolic capabilities to degrade xenobiotics used in industrial applications and bioremediation [64]. Candidate UV-20, identified as *P. nicotinovorans*, has been not only described as having nitrogen-fixing capacity [65], but it is also capable to degrade nicotinic acid, an essential growth factor for *E. amylovora* [66]. This ability could be decisive to confirm its antagonistic capacity against this phytopathogen. However, to date, only the biocontrol potential of *Paenarthrobacter ureafaciens* has been previously evaluated and reported [59].

*Pseudomonas* species stand out for having great metabolic versatility and a broad spectrum of activities related to pathogen biocontrol, such as antibiotic and hydrolase production, and plant growth promotion such as nitrogen and other nutrient fixation [67]. Even though *P. moraviensis* has not been described as a biocontrol agent to date, its phosphate-solubilizing ability to promote plant growth and the results obtained in this work may make isolate UV-30 a suitable candidate for antagonism against *E. amylovora* [68].

The efficacy of some antagonistic bacteria for the control of fire blight in detached loquat and/or pear fruit has been demonstrated in recent studies with plant-associated bacterial species [40] and, more recently, with a strain of *Priestia megaterium* isolated from a pear orchard [56]. However, neither *Paenarthrobacter aurescens*, *P. nicotinovorans* nor *Pseudomonas moraviensis* from fire blight-free Mediterranean environments have been described so far as potential biocontrol agents of *E. amylovora*. Notwithstanding, in order to evaluate the potential of the selected candidates as biocontrol agents against fire blight, further *in vivo* studies would be needed to assess their potential to be used in biocontrol under field conditions.

## 4. Conclusions

The present study concludes that the selected bacterial candidates have demonstrated *in vitro* antagonistic ability against *E. amylovora* through nutrient competition and the production of antimicrobial substances in some cases. Results of antagonism assays on immature fruits of two host species susceptible to *E. amylovora* revealed the ability of some of them to reduce and delay the onset of fire blight symptoms, with several of them showing hydrolytic and plant growth-promoting activities related to biocontrol potential. These antagonistic bacteria could contribute to providing or improving new, safer, and more environmentally friendly biological control strategies against fire blight disease.

## Figures and Tables

**Table 1:** Host plant, origin, and year of isolation of the *Erwinia amylovora* strains used in this study.

Strain	Host Plant	Origin	Year of Isolation	Reference
CFBP ^1^ 1430	*Crataegus oxyacantha*	France	1972	[37]
IVIA ^2^ 1554	*Crataegus* sp.	Segovia (Spain)	1996	[38]
IVIA 1614.2	*Pyracantha* sp.	Segovia (Spain)	1998	[39]
IVIA 1892.1	*Pyrus communis*	Guadalajara (Spain)	1998	[38]

^1^ CFBP: Collection Française de Bactèries Phytopathogènes ^2^ IVIA: Instituto Valenciano de Investigaciones Agrarias.

**Table 2:** *In vitro* antagonistic activity of the potential candidates against *Erwinia amylovora* strains.

Candidates	Status	*E. amylovora* Strains			
		CFBP 1430	IVIA 1554	IVIA 1614.2	IVIA 1892.1
UV-9	Active	−	+	+	++
	Inactive	−	+	+	++
UV-12	Active	−	+++	++	+
	Inactive	−	−	−	−
UV-17	Active	+	+++	++	+
	Inactive	−	+++	+	−
UV-18	Active	++	+++	+++	+++
	Inactive	++	+++	+++	+++
UV-19	Active	++	+++	+	+++
	Inactive	+	+++	+	++
UV-20	Active	+	+++	++	++
	Inactive	−	++	+	+
UV-30	Active	−	+++	++	++
	Inactive	−	−	−	−
UV-41	Active	++	++	++	++
	Inactive	++	++	++	++
UV-59	Active	−	−	++	+
	Inactive	−	−	++	+
UV-60	Active	−	−	++	−
	Inactive	−	−	++	−

(−): No antagonistic activity; (+): low antagonistic activity (*E. amylovora* streak ≥ 41 mm); (++): moderate antagonistic activity (*E. amylovora* streak from ≤40 to ≥21 mm); (+++): strong antagonistic activity (*E. amylovora* streak ≤ 20 mm).

**Table 3:** Exoenzymatic activities and nitrogen-fixing activity of the potential biocontrol candidates against *Erwinia amylovora*.

Bacterial Isolate	Exoenzymatic Activity ^1^							Nitrogen-Fixing Activity ^2^	
	Protease		Lipase		Amylase	Cellulase	DNase		
	48 h	96 h	48 h	96 h	96 h	96 h	96 h	48 h	96 h
UV-9	+++	+++	+++	+++	+++	+++	+	−	−
UV-12	++	++	−	++	−	−	+	+	+
UV-17	−	++	−	−	+++	−	+	+	+
UV-18	++	+++	−	−	+++	−	+	−	+
UV-19	++	+++	−	−	+++	−	+	−	+
UV-20	−	++	−	−	+++	−	−	+	+
UV-30	−	−	−	++	−	−	−	+	+
UV-41	−	++	+	+++	+++	+++	−	−	−
UV-59	−	−	−	−	+++	−	+	−	−
UV-60	−	−	−	−	+++	−	+	−	−

^1^ Quantitative results in terms of enzymatic activity halo diameter (mm): (−) no activity; (+) weak activity, halo ≤ 10 mm; (++) moderate activity, halo from ≥ 11 to ≤ 20 mm; (+++) strong activity, halo ≥ 21 mm. ^2^ Qualitative results in terms of presence/absence of bacterial growth: (+) bacterial growth due to nitrogen-fixing activity; (−) absence of activity and growth.

**Table 4:** Identification of candidates with the strongest antagonistic activity against *Erwinia amylovora*.

Strain Code	Identification (Matched Pattern)	Score Value ^1^
UV-12	*Bacillus simplex* CS 206_1aI BRB	1.91 (+)
UV-17	*Paenarthrobacter aurescens* DSM 20116T	2.34 (+++)
UV-20	*Paenarthrobacter nicotinovorans* DSM 420T	1.86 (+)
UV-30	*Pseudomonas moraviensis* DSM 16007T	2.39 (+++)

^1^ Scale: (−) identification not possible; (+) probable identification at genus level; (++) secure identification at genus level, probable identification at the species level; (+++) high probability of identification at the species level.

## Data Availability

Not applicable.
